# Holocaine

**Published:** 1898-09-10

**Authors:** 


### HOLOCAINE.

Of the several now drugs which have been introduced into ophthalmic practice as substitutes for cocaine, holocaine is specially deserving of notice from the fact that it appears to act purely as an anæsthetic, and not to interfere with the mechanism of the eye. Dr. Hinshelwood, speaking at Edinburgh, said that the hydrochlorate of chlorohydrate of holocaine takes the form of white needles, slightly soluble in cold water but more readily in warm. Its aqueous solution is neutral and undergoes no change on prolonged boiling. A 1 per cent. solution produces complete anæsthesia in from 15 to 30 seconds after instillation. There is no alteration of the size of the pupil or of the tension of the eye, nor is there any disturbance of the accommodation, and the corneal epithelium is not changed in the slightest, but retains its normal appearance. In fact, it seems to have no other effect upon the eye than rendering it anæsthetic, and thus it possesses an advantage over cocaine in several directions. Moreover, from the rapidity of its action there is much saving of time where many eyes have to be examined. The disadvantage is that it is difficult to get a clear solution, and that when made it will not keep long.

